# Evaluation of High-Temperature Performance of Hungarian Bituminous Binders Using the BTSV Method

**DOI:** 10.3390/ma19102012

**Published:** 2026-05-12

**Authors:** Szabolcs Rosta, László Gáspár

**Affiliations:** 1 Multidisciplinary Technical Scientific Doctoral School, Széchenyi István University, 9026 Győr, Hungary; rosta.szabolcs@dunaaszfalt.hu; 2 Department of Transport Construction and Water Management, Széchenyi István University, 9026 Győr, Hungary

**Keywords:** BTSV (Bitumen Typisierungs Schnell Verfahren), SBS-modified bitumen, chemically stabilized rubber-modified bitumen, Soxhlet extraction

## Abstract

In Europe, bitumen classification has traditionally relied on empirical tests, namely penetration and the Ring and Ball softening point, originally developed for unmodified binders and considered insufficient for modern modified binders. As an alternative, a rheology-based method, the Bitumen Typisierungs Schnell Verfahren (BTSV) rapid bitumen categorization method, has been developed in Germany to characterize high service temperature performance, with performance requirements introduced in 2025 in the German specifications. In this study, the performance of five bitumen types commonly used in Hungarian road construction was investigated using the BTSV method. During testing, the softening temperature corresponding to a rheological threshold value of G* = 15.0 kPa (T_BTSV_) and the phase angle (δ_BTSV_) were determined. T_BTSV_ is defined as the temperature corresponding to G* = 15 kPa, representing the softening state, while δ_BTSV_ reflects the viscoelastic balance between elastic and viscous behaviour. The objective of this study is to evaluate the high-temperature performance of commonly used Hungarian bituminous binders using the BTSV method and to compare the results with traditional empirical parameters and German classification systems. A total of 137 binder samples from production control were tested and analysed, including paving-grade, SBS-modified, and chemically stabilized rubber-modified binders. Statistical evaluation included mean values and 95% confidence intervals. For rubber-modified bitumens, the recoverable, insoluble rubber content was determined using the Soxhlet extraction method. Based on the results, it can be concluded that with increasing rubber content, the T_BTSV_ value shows an increasing trend, while the δ_BTSV_ value decreases. As discussed in the paper, a strong linear relationship was observed between the investigated parameters in the T_BTSV_–δ_BTSV_ diagram, with a coefficient of determination of R^2^ = 0.99.

## 1. Introduction

The requirements for bitumens and bituminous binders are primarily determined using physical properties observed in various temperature ranges. The most common bitumen classification systems worldwide are based on empirical tests, which are based on direct experience, observation or experiments, such as needle penetration [1] in the medium in-service temperature range and the Ring and Ball softening point [2] test, which characterizes high in-service temperatures.

The specification framework developed by the European Union (EU) does not define specific property limit values for bitumen categories. Instead, Member States may determine the property requirements for bitumen types within their own competence, considering local climatic and traffic conditions [3,4].

Within the EU Member States, no significant differences are observed in the Ring and Ball softening point values assigned to individual penetration classes of paving-grade bitumen. In contrast, significant differences can be identified in the case of modified bitumens. As an example, different softening point requirements were specified for Hungarian (PmB 45/80-65) and German (PmB 45/80-50 A) PmB types, while the penetration value remains identical [5,6].

Originally, these empirical tests were developed for the characterization of unmodified bitumens. Recent research works have shown several shortcomings and inaccuracies of this test method, especially due to the increasing complexity of the materials and their modification techniques. These types of tests do not always provide sufficiently accurate or consistent characterization of rheological behaviour and performance of polymer-modified bitumens or aged binders from reclaimed asphalt pavement (RAP) [7,8,9].

In the United States (US), traditional empirical tests such as penetration or the Ring and Ball softening point are no longer used to classify bituminous binders. These methods have been replaced by the Superpave Performance Classification (PG) system, introduced as a result of the Strategic Highway Research Program (SHRP) implemented between 1987 and 1992 [10], which evaluates the behaviour of binders based on rheological parameters [11]. The system uses a dynamic shear rheometer (DSR) to determine the high in-service temperature performance of the binder [12]. The high-temperature PG class is determined using the G/sinδ* parameter calculated from the relationship between the complex shear modulus (G*) and the phase angle (δ); both parameters are determined during the DSR test. The critical temperature is determined based on the limits of G/sinδ ≥ 1.00 kPa (at a frequency of 1.59 Hz/s) measured on an unaged binder and G/sinδ ≥ 2.20 kPa measured on an aged binder, where the aging is carried out by the Rolling Thin Film Oven Test (RTFOT) procedure [13]. The purpose of the specified limits is to ensure that the selected binder exhibits adequate resistance to permanent deformation at high in-service temperatures.

However, several studies have shown that the G/sinδ* rutting parameter is not always able to adequately predict the performance of polymer-modified binders and often shows poor correlation with the behaviour of asphalt mixtures [14,15,16]. As a result, in 2015, the US introduced the Multiple Stress Creep and Recovery (MSCR) test as a standard test method to determine the high in-service temperature performance of modified binders [17].

However, there are also some limitations in the application of the MSCR test. According to some researchers, DSR testing cannot apply the block-like stress signal completely without delay in certain cases for short creep periods [18,19]. Therefore, it becomes necessary to introduce more modern testing methods that are based on the science of rheology and allow a better understanding of the viscoelastic behaviour of modern binders.

This research work also presents the testing of a special rubber-modified bitumen product, which has been used in Hungary as a bituminous binder for asphalt mix production since 2013. The development of the European patent for a chemically stabilized rubber bitumen (CSRB) can be considered a significant qualitative step forward in the field of rubber-modified bitumens [15,20]. The favourable results of extensive laboratory tests carried out earlier [21] and the positive outcomes of long-term in situ performance evaluation on a number of trial sections subject to heavy traffic [22] resulted in an increasingly important role of this environmentally friendly technique in Hungarian road technology selection [23,24,25]; therefore, this binder was investigated in depth in this paper. The objective of this study is to evaluate the high-temperature performance of commonly used Hungarian bituminous binders using the BTSV method and to compare the results with traditional empirical parameters and German classification systems.

## 2. Materials and Methods

### 2.1. Background

Various authors have previously determined the softening point of unmodified binders based on viscosity and oscillation measurements at their Ring and Ball softening point temperatures [9,26]. However, when examining modified binders, no reliable correlation was identified between the rheological parameters and the empirical softening point.

Alisov [27] showed that at the softening point temperature, polymer-modified binders had a significantly lower complex shear modulus (up to G* = 2.563 kPa).

In contrast, for conventional paving-grade bitumens, the complex shear modulus at the softening point temperature is typically around G* ≈ 15 kPa. The highest value was measured for the paving-grade bitumen B50/70, reaching G* = 15.682 kPa, while the lowest value was only approximately 2.563 kPa for polymer-modified binders.

A difference of about 13 kPa between the two binder types corresponds to a temperature difference of about 12.5 °C. This range illustrates the approximate rheological equivalence of the material state at the softening point temperature. This means that the softening point can only approximately characterize the equivalent rheological material state.

The DSR apparatus and test method offer a reliable experimental alternative for determining the softening state of a bituminous binder.

Based on the test results of the unmodified binder, the softening point temperature represents a material state characterized by a complex shear modulus of G* = 15 kPa, which was also confirmed by other exploratory studies [26]. However, in the case of modified binders, the definition of the softening state based on the empirical softening point is not sufficient [27]. Despite this inconsistency, the threshold value of G* = 15 kPa is also applied to polymer-modified bitumens. The advantages of using the DSR include the generation of rheological data, small material volumes for testing, and the simplicity and speed of the test procedure. By determining the isomodulus temperature corresponding to a shear modulus of 15 kPa, the material behaviour of the binder at elevated temperatures can be suitably characterized.

**Figure 1:**
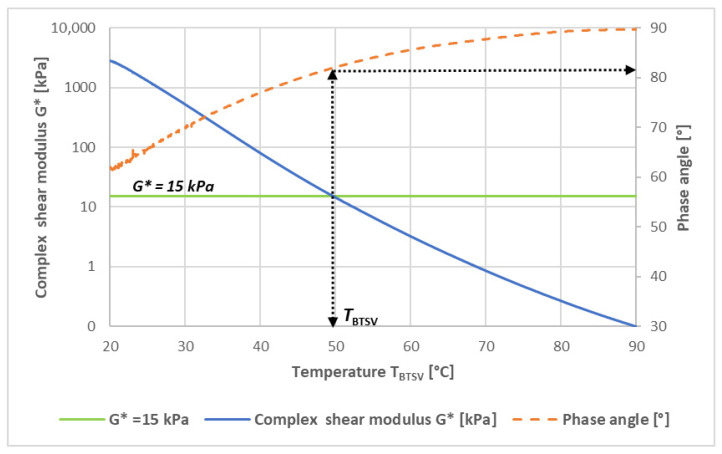
Typical curves of the complex shear modulus and phase angle as a function of test temperature [28].

Considering the above, a simple method, the Bitumen Typisierungs Schnell Verfahren (BTSV) [28], also known as the rapid bitumen categorization method, was developed at the University of Braunschweig in Germany to characterize bituminous binders. A constant oscillatory shear stress of 500 Pa at a constant frequency of 1.59 Hz/s is applied to a parallel plate binder sample of a standard 25 mm diameter, while the temperature is gradually increased from 20 °C to 90 °C, with a speed of ΔT = 1.2 °C/min. The test normally takes about 60 min. With this new German procedure, the softening state is characterized by two main parameters, the softening temperature (T_BTSV_) and the phase angle (δ_BTSV_), which are taken when the sample reaches a G* = 15.0 kPa complex shear modulus (Figure 1).

In 2025 the national specification [6] was issued within the framework of the German road specification system (FGSV), which defines the quality and testing requirements for paving-grade bitumens and polymer-modified bitumens.

The document is based on the European bitumen framework [3,4] but supplements with more detailed physical, rheological, and aging parameters regarding temperature and load-dependent behaviour. In addition to the traditional consistency characteristics (e.g., penetration, softening point), the regulation also includes the parameters determined using the BTSV procedure, T_BTSV_ and δ_BTSV_. This makes Germany the first European country to include these parameters with limits in its national bitumen delivery system.

The BTSV test procedure has not yet been included in the European standardization framework; however, this work is underway, and the regulatory body has been working on the definition of a performance-based bitumen classification since the 2010s. In 2020, the regulatory body only envisaged performance-based classification for modified bitumens, and the framework specification [29] included T0 and δ0 as the two parameters to determine the viscoelastic behaviour, tested on unaged binders. In the latest draft titled “Bitumens and bituminous binders—Complementary performance-related specification framework” [30], T1 and δ1 are included as parameters tested on RTFOT short-term aged binders [31]. In the absence of harmonized EU regulations on this topic, BTSV has not appeared yet as parameters to be included in Hungarian technical specifications; the work presented in this paper is the first comprehensive study that discusses this issue in Hungary in a systematic manner.

**Figure 2:**
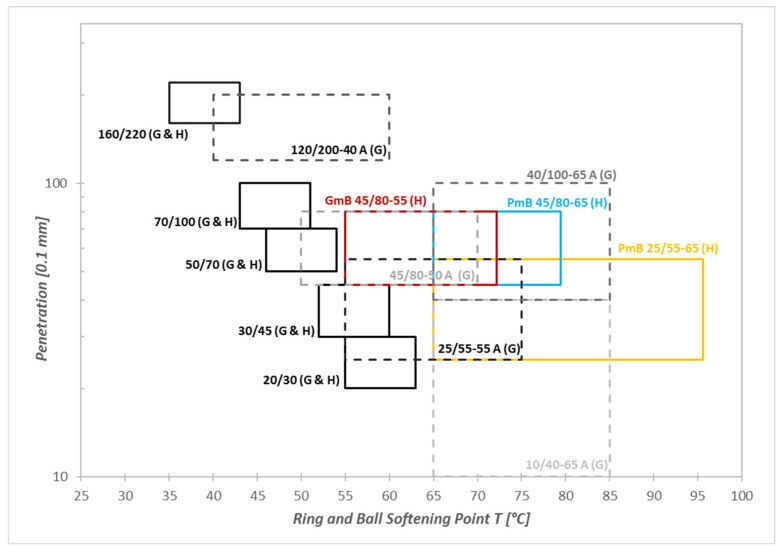
Softening point and penetration limits of Hungarian (H) and German (G) bitumens.

Figure 2 shows that there are detectable differences in terms of empirical characteristics between the limits of the Hungarian (coloured lines) and the German polymer-modified bitumens (grey dashed lines). For example, for the German PmB 25/55-55-A type, the Hungarian regulation sets a higher minimum requirement of 65 °C for the Ring and Ball softening point, while the German specification stipulates a value of 55 °C. This difference arises from the fact that the European framework [4] allows Member States to choose the property limit values belonging to the bitumen classes in their national specifications—considering local climatic conditions, traffic loads and pavement construction practice. The upper limit of the softening point of German PmB bitumens was determined based on the maximum permissible values of bitumen recovered from asphalt, as determined by the regulation in [32]. In Hungary, there is no such upper limit for the empirical softening point. Therefore, for the sake of comparability, a set of xy production samples was tested for the penetration and softening point, and the arithmetic mean (X) and standard deviation (SD) were calculated for this population. Given that the set was large enough, normal distribution could be assumed, and the limits of the population could be established with 95% confidence by two standard deviations added to the mean ($\bar{X}$ + 2SD). In this case, the upper limit of the CSRB 45/80-55 binder was determined to be 73 °C, while the upper limit for the PmB 45/80-65 binder was 80 °C. These values are 3 °C and 10 °C higher, respectively, than the upper limit specified for the German PmB 45/80-50 binder. For the PmB 25/55-65 binder, an upper limit of 95 °C was obtained, which is approximately 20 °C higher than that of the corresponding German binder with the same penetration grade (25/55-65).

In contrast, regarding paving-grade bitumens, described by penetration according to EN 12591 [3] and indicated by a continuous black line, there is no difference between the Hungarian and German product standards. The requirements of the harmonized European standard system apply uniformly to these binder classes; therefore, the limits of the penetration and Ring and Ball softening point are equally applied.

### 2.2. Materials

The objectives of the research work were to sample 5 different types of bitumen most frequently used in Hungarian asphalt road construction. The samples were taken at various asphalt mixing plants of the Duna Group between 2022 and 2023. Each sample represents approximately 100 tons of bitumen and was collected in accordance with the standardized sampling procedure [33], ensuring representative sampling of the material. This corresponds to approximately one week of production at the asphalt mixing plant. Considering the total number of samples, the monitored bitumen quantity is equivalent to approximately one year of asphalt production. Across the 5 bitumen types, a total of 137 bitumen samples were taken: two normal (unmodified) paving-grade bitumen samples—B50/70 and B70/100—and three modified bituminous binders—two of them SBS-modified (PmB 25/55-65, and PmB45/80-65) and one chemically stabilized rubber-modified GmB 45/80-55 bitumen.

In addition to the conventional empirical properties (needle penetration and Ring and Ball softening point), two other parameters obtained from the BTSV test, the softening temperature (T_BTSV_) and the corresponding phase angle (δ_BTSV_), were measured. The individual results were not tabulated. Table 1 summarizes the statistical measures of the tested properties.

## 3. Results

### 3.1. Relationship Between T_BTSV_ Temperature and Ring and Ball Softening Point

The high in-service temperature of bituminous binders is the upper limit of the application temperature range, when the asphalt mix manufactured with the relevant bitumen still resists significant plastic deformation. This characteristic is especially important during summer, combined with high-volume heavy traffic, since the consistency of bitumen is no longer able to maintain its shape above a certain temperature and flows. Based on previous research, the empirical softening point temperature of unmodified bitumens is approximately the same as the temperature at which the complex shear modulus equals 15 kPa, which is also the T_BTSV_. This relationship is not always valid for modified bitumens, since these materials are more elastic and they likely recover their shape after deformation. This elastic behaviour is described by a lower phase angle (δ_BTSV_), indicating that their behaviour is less viscous and more elastic. For this reason, the value G* = 15 kPa is not reached at the same temperature as the empirical Ring and Ball softening point [34].

**Figure 3:**
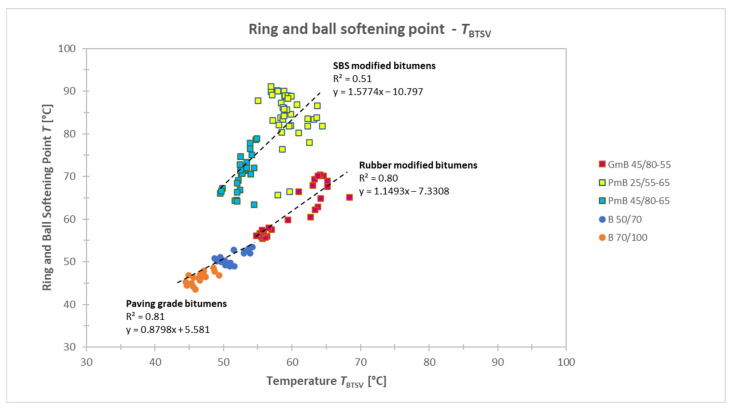
Relationship between Ring and Ball softening point and T_BTSV_ for SBS-modified, rubber-modified and paving-grade bitumens.

Figure 3 illustrates the relationship between the softening point of bitumens and the characteristic temperature (T_BTSV_, °C) determined by the BTSV method for tested modified and paving-grade bitumens.

In general, the strength of correlation is often classified into several intervals based on the coefficient of determination (R^2^). Accordingly, weak (R^2^ < 0.3), moderate (0.3 ≤ R^2^ < 0.7), and strong (R^2^ ≥ 0.7) relationships can be distinguished.

When examining the paving-grade bitumen types (B 50/70 and B 70/100), a significant nearly linear relationship can be observed between the T_BTSV_ and the empirical softening point. The coefficient of determination, R^2^ = 0.81, indicates a strong linear correlation between the two parameters. These findings suggest that, for paving-grade bitumens, the T_BTSV_ value increases proportionally with the increasing Ring and Ball softening point. This behaviour may be attributed to the fact that the rheological properties of these bitumens are primarily determined by the temperature-dependent viscoelastic characteristics of the base bitumen.

In contrast, for modified bitumens (PmB and GmB), there is no significant linear relationship between the T_BTSV_ and the Ring and Ball softening point (R^2^ = 0.06). For these binders, the softening point does not reflect exclusively the properties of the base bitumen but is significantly influenced by the presence of the modifiers. The results of the samples belonging to the GmB 45/80-55 type showed almost parallel regression line to the T_BTSV_–Ring and Ball softening point relationship or practically followed the regression line of paving-grade bitumens. Based on this, the separate statistical evaluation of polymer-modified bitumen (PmB) and rubber-modified bitumen (GmB) became justified, as their combined analysis would mask the distinct behaviours of the individual binder types.

For the GmB 45/80-55 binder, the coefficient of determination was R^2^ = 0.80, which is very similar identified for unmodified bitumens. The value of the coefficient of determination for polymer-modified bitumens (PmB) was 0.51. This indicates a medium-strength relationship and cannot be considered reliably linear. Based on the rheological behaviour (G* ≈ 15 kPa, δ) it was found that the classical empirical parameters alone are not sufficient enough to accurately describe the viscoelastic behaviour of bituminous binders.

### 3.2. BTSV Parameters

**Figure 4:**
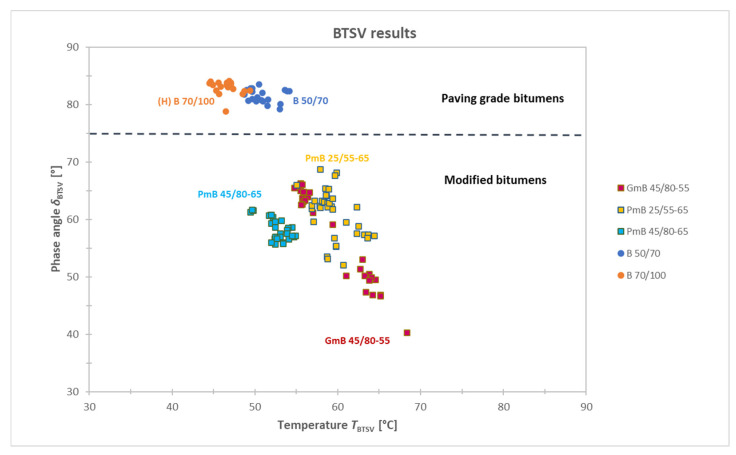
Summary of the BTSV results: the phase angle δ_BTSV_ and T_BTSV_ measured at G* = 15 kPa.

The temperature T_BTSV_ [°C] and the phase angle δ_BTSV_ [°] of all 137 samples tested are shown in Figure 4. From this graphical representation of the results, two large groups of paving-grade bitumens and modified bitumens can be clearly distinguished, and an arbitrary horizontal dotted line is provided on the chart.

The polymer modification primarily affects the elastic component of the viscoelastic behaviour of the bitumen, and the extent of the elastic part is expressed by the phase angle. The greater the degree of modification, the lower the δ_BTSV_ value [27].

The threshold value of δ_BTSV_ = 75° was defined based on the evaluation of the boundary values of the investigated materials. Specifically, the highest phase angle observed for polymer-modified binders and the lowest phase angle measured for paving-grade bitumens were compared. The difference between these values was approximately 10°, and, therefore, a practical, rounded threshold value was introduced, resulting in δ_BTSV_ = 75°.

Accordingly, bitumens with a δ_BTSV_ > 75° are predominantly viscous in character based on their rheological behaviour and can be considered as paving-grade bitumens. Binders with a δ_BTSV_ ≤ 75°, on the other hand, have a significant elastic component, indicating the presence of some modification.

**Figure 5:**
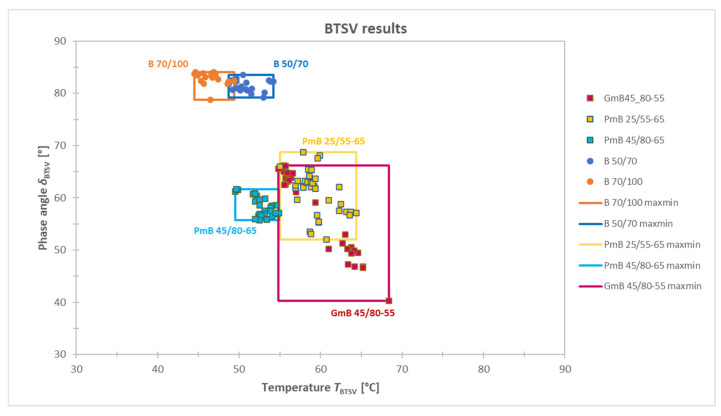
Maximum and minimum limits of various binders described by δ_BTSV_ and T_BTSV_.

Following further analysis, the characteristic ranges of the individual bitumen types were also determined as summarized in Figure 5. The range limits of each bitumen type, defined by their minimum and maximum values, are represented as bounding frames and illustrated using coloured rectangles. The comparison of these characteristic limits shows that partial overlaps can be observed between various bitumen types. This overlap may indicate that under certain temperature and loading conditions, different types of binders may exhibit similar rheological properties, leading to similar performance at high in-service temperatures.

Based on the above analysis, it was found that the two paving-grade bitumen types (B 50/70 and B 70/100) and the two polymer-modified binders (PmB 25/55-65 and PmB 45/80-65) form clearly distinct and statistically different product groups. The rubber-modified bitumen (GmB 45/80-55) is considered a separate product group, as a significant scatter of values can be observed in terms of both the phase angle and the softening temperature. Since the BTSV test was conducted in accordance with the test method, it is unlikely that the scatter in the test data is coming from test errors or variable test conditions. A possible explanation for this phenomenon is to be found in the specific multiphase structure of rubber modified bitumen. During the sampling process at the time of asphalt production, there is a possibility that the binder sample was not completely homogeneous, given the nature of rubber-modified binders, as they tend to segregate. This may have resulted in heterogeneous rheological properties of the tested samples. Previous research work has already proven that the type of modifying substance and its amount have a significant effect on the BTSV result and the degree of modification has a significant effect on the phase angle [27].

**Figure 6:**
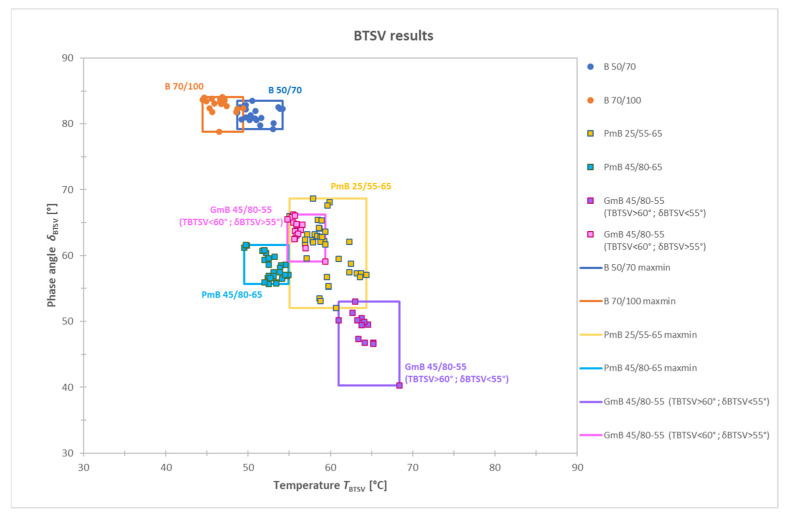
Summary of BTSV results, with GmB 45/80-55 bitumen divided into two groups.

Based on the results of the tested rubber-modified bitumen samples, the GmB 45/80-55 type can be split into two clearly distinguishable subgroups, which have different characteristics (Figure 6). The first subgroup is characterized by a lower softening temperature (T_BTSV_ < 60 °C) and correspondingly higher phase angle (δ_BTSV_ > 55°). This behaviour indicates a higher viscous component, i.e., the resistance of the material to deformation, and a less elastic nature in the examined temperature range. The second subgroup, on the other hand, shows a higher softening temperature (T_BTSV_ > 60 °C) and a lower phase angle (δ_BTSV_ < 55°). A lower δ_BTSV_ value indicates a higher elastic component. Based on these rheological characteristics, it is likely that the proportion of rubber crumb in the latter sample group was higher.

**Figure 7:**
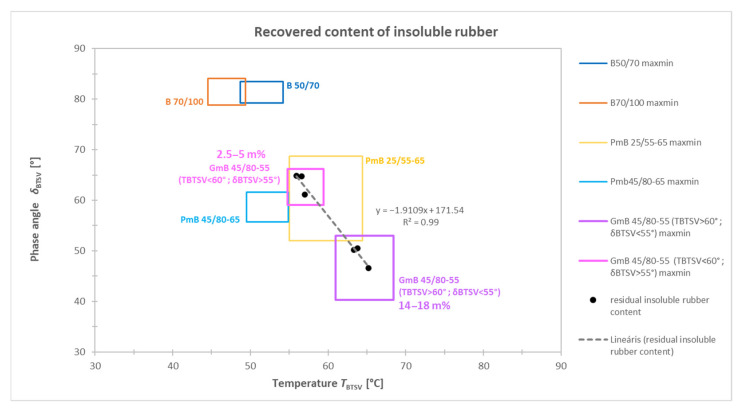
Effect of increasing recoverable rubber crumb content on T_BTSV_.

In order to verify the above hypothesis, the recoverable, insoluble rubber content was determined by Soxhlet extraction using the Australian test method [35]. From each group, three samples were randomly selected. The test results indicated that samples with a higher phase angle had a rubber content of only 2.5–5.0 wt%, while the lower phase angle corresponded with a significantly higher rubber content of around 14–18 wt% (Figure 7).

The results indicate that an increasing rubber content tends to be associated with the level of modification and corresponding changes in rheological behaviour (R^2^ = 0.99): the presence of a larger amount of rubber crumb resulted in an increased elastic property, which is manifested in a decrease in the phase angle and higher T_BTSV_ values. This confirms that the amount of rubber content is a determining parameter in the development of the high-temperature viscoelastic properties of CSRB-s.

### 3.3. Categorization of the Tested Bitumens According to BTSV

**Figure 8:**
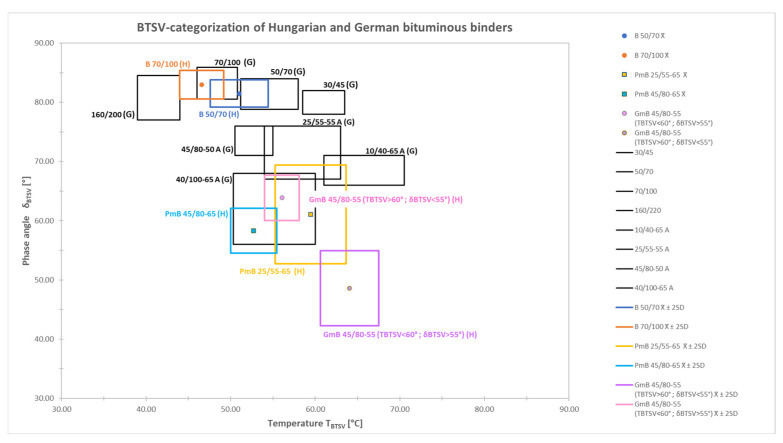
BTSV categorization of the most common Hungarian (H) and German (G) bitumen types as a function of phase angle (δ_BTSV_) and temperature (T_BTSV_).

To characterize the bitumens, the T_BTSV_–δ_BTSV_ parameter pair was again represented graphically. As before, Figure 8 shows the characteristic limits of each bitumen category, while each type is marked with a representative average point ($\bar{x}$) in Figure 8. In order to make the data more accurately comparable, the outliers (extreme values) were filtered using a statistical method.

It is commonly accepted in pavement engineering that key bitumen properties (e.g., penetration, softening point, and rheological parameters) can be reasonably approximated by normal or near-normal distributions, particularly under quality-controlled production conditions (e.g., Tarefder et al., 2010 [36]). Accordingly, a normal (Gaussian) distribution was assumed as an approximation for the statistical evaluation of the data. The interval “mean ± 2 standard deviations ($\bar{x}$ ± 2SD)” was applied, covering approximately 95% of the data. This approach allowed the exclusion of outliers from the categorization, while retaining a representative part of the dataset. In Figure 8, the BTSV method categorization of bitumens used in Germany is marked with black lines as a function of the softening temperature (T_BTSV_) determined at G* = 15 kPa and the associated phase angle (δ_BTSV_).

The resulting (colourful) areas, bounded by $\bar{x}$ ± 2SD, represent categories derived from Hungarian tests plotted against the (black) German classification fields, enabling direct comparison of the two product systems.

For the investigated Hungarian paving-grade bitumens (50/70 and 70/100), it can be observed that the phase angle ranges show a high level of conformity (≈95%) with the corresponding German bitumen specifications of equal penetration grade. However, the average T_BTSV_ values of the Hungarian B70/100 (H) and B50/70 (H) bitumens are lower than those of their German counterparts, with differences of approximately 2 °C and 4 °C, respectively.

In the case of PMB 25/55, the majority of Hungarian samples (≈97%) occurred within the German T_BTSV_ range. In contrast, a significant deviation is observed in terms of the phase angle: only three samples fall within the German boundaries, while the remaining samples (≈91%) exhibit substantially lower values. The Hungarian PmB 25/55-65 (H) shows an average phase angle approximately 10° lower than that of the German PmB 25/55-55 A (G) class.

For the PMB 45/80 category, the TBTSV threshold values differ by less than 1 °C, and approximately 90% of the investigated PmB 45/80-65 (H) samples are based within the German PmB 45/80-50 A (G) class. A difference of approximately 15° is observed in the phase angle, with all Hungarian measurements yielding lower values.

In the case of polymer-modified categories, the Hungarian binders exhibit significantly lower phase angle ranges, suggesting a higher level of modification.

The GmB 45/80-55 (H) type shows a fundamentally different behaviour compared to the German classes presented in the figure; consequently, a direct comparison is not appropriate.

## 4. Discussion

The average temperature corresponding to the G* = 15 kPa value of the Hungarian paving-grade bitumens B 70/100 and B 50/70 occurs in a lower range for the Hungarian samples. As a result, the category boundaries of the Hungarian bitumens shift to the left in the T_BTSV_–δ_BTSV_ diagram compared to the boundaries of the German bitumens. For the paving-grade bitumens, the phase angle ranges show a large overlap with those of the German bitumens from the same penetration class. This indicates that in terms of the δ_BTSV_ parameter, the rheological behaviour of the Hungarian and German paving-grade bitumens is similar, i.e., there is no significant difference in the viscoelastic characteristics between these bituminous binders.

As for the polymer-modified bitumens (PmB), a marked difference can be observed in the phase angle values.

German PMBs belonging to the same penetration category (e.g., 45/80-50 A and 25/55-55 A) typically have a δ_BTSV_ range above 65° and close to around 70°; therefore, a more moderate modification can be assumed for these products. In contrast, the tested Hungarian PMBs (PmB 25/55-65 and PmB 45/80-55) show an average phase angle below 65°. Since the lower δ_BTSV_ value indicates a larger elastic component, it is likely that the tested Hungarian-made PmB binders have a higher SBS polymer content and/or more intensive modification.

It is worth examining the behaviour of the GmB 45/80-55 CSRB separately. One subgroup of the samples (T_BTSV_ < 60 °C; δ_BTSV_> 55°) showed similar properties to the German 40/100-65 A type bitumen based on its rheological parameters. In contrast, the other CSRB subgroup (T_BTSV_> 60 °C; δ_BTSV_ < 55°) is completely different from the other binder groups compared to both the Hungarian and the German bitumen types. This separation can be characterized by a higher T_BTSV_ temperature and a lower phase angle, which, due to the rubber content, indicates a more significant elastic behaviour and a more intense modification effect.

## 5. Conclusions

The results of this study indicate that the BTSV method can serve as a complementary testing to conventional empirical tests (needle penetration and Ring and Ball softening point), enabling a more detailed and performance-related characterization of bituminous binders. Also, the dynamic shear rheometer (DSR) requires a small sample size, while more accurate and reproducible results can be obtained with this equipment type. In addition, the method provides a significantly more detailed characterization of the high-temperature performance of various binder types.

Among the BTSV parameters, the introduction of the phase angle (δ_BTSV_) represents a major advancement in the evaluation of bituminous binders. This parameter enables a quantitative assessment of the differences in rheological behaviour between binders with similar softening points.

Based on the evaluation of a large dataset of production samples, characteristic parameter ranges and limit values for the most commonly used bitumen types in Hungary were determined. These results contribute to the establishment of a performance-based classification system and provide a basis for future specification development.

The comparison between Hungarian and German modified bitumens revealed notable differences in their rheological behaviour. While similar T_BTSV_ ranges were observed, Hungarian polymer-modified binders generally exhibited lower phase angle (δ_BTSV_) values, indicating a higher elastic component and a more pronounced modification effect. In contrast, German binders of comparable penetration classes showed higher δ_BTSV_ values, suggesting a moderate level of modification.

In the case of Hungarian rubber-modified bitumen, a significant variability in rheological properties was observed, which could be attributed to differences in rubber content. The results confirmed that increasing rubber content leads to higher T_BTSV_ values and lower δ_BTSV_ values, indicating an enhanced elastic response. This highlights the strong influence of rubber modification on binder performance and underlines the importance of considering material composition in rheological classification. This difference highlights the importance of rheological parameters in distinguishing binder performance beyond traditional classification systems.

Overall, the DSR-based BTSV method provides a reliable and efficient tool for the performance-oriented characterization of bituminous binders and offers significant advantages over traditional empirical testing methods.

## Figures and Tables

**Table 1:** Bitumen sample sizes and statistical characteristics of parameter values.

Sample Type	Sample Size	Parameter	Avg.	St. Dev.	Min.	Max.
B 50/70	20	Softening point [°C]	50.77	1.38	49.00	53.40
		Penetration [0.1 mm]	58.15	5.40	52.00	69.00
		T_BTSV_ [°C]	51.01	1.72	48.70	54.20
		Phase angle, δ [°]	81.48	1.15	79.20	83.50
B70/100	20	Softening point [°C]	46.25	1.28	43.50	48.60
		Penetration [0.1 mm]	84.00	5.95	73.00	93.00
		T_BTSV_ [°C]	46.57	1.31	44.50	49.40
		Phase angle, δ [°]	82.95	1.21	78.80	84.10
GmB 45/80-55	29	Softening point [°C]	61.22	5.50	55.50	70.40
		Penetration [0.1 mm]	45.13	3.67	39.10	49.20
		T_BTSV_ [°C]	59.64	4.26	54.80	68.40
		Phase angle, δ [°]	57.03	8.12	40.30	66.20
PmB 25/55-65	38	Softening point [°C]	84.28	5.65	65.60	91.05
		Penetration [0.1 mm]	42.21	6.22	29.50	52.00
		T_BTSV_ [°C]	56.57	4.05	51.90	64.40
		Phase angle, δ [°]	59.46	2.10	55.05	68.80
PmB45/80-65	30	Softening point [°C]	70.75	4.36	63.35	78.90
		Penetration [0.1 mm]	52.24	4.98	40.85	59.00
		T_BTSV_ [°C]	52.72	1.37	49.50	54.90
		Phase angle, δ [°]	58.29	1.89	55.70	61.60

## Data Availability

The original contributions presented in this study are included in the article. Further inquiries can be directed to the corresponding author.

## Abbreviations

The following abbreviations are used in this manuscript:

DSR	Dynamic Shear Rheometer
BTSV	Rapid Bitumen Categorization Method
RAP	Reclaimed Asphalt Pavements
US	United States
PG	Superpave Performance Classification
SHRP	Strategic Highway Research Program
RTFOT	Rolling Thin Film Oven Test
MSCR	Multiple Stress Creep and Recovery
CSRB	Chemically Stabilized Rubber Bitumen
AS	Standards Australia
EN	European Norm
ASTM	American Society for Testing and Materials
SD	Standard Deviation
$\bar{X}$	Arithmetic Mean
